# Using systematic reviews to identify research gaps - a case study: mIBG for the treatment of neuroblastoma in children

**DOI:** 10.1186/1745-6215-14-S1-P126

**Published:** 2013-11-29

**Authors:** Jayne Wilson, Jenny Gains, Veronica Moroz, Mark Gaze, Keith Wheatley

**Affiliations:** 1 CRCTU, University of Birmingham, West Midlands, UK; 2 MRC MHTMR, University of Birmingham, West Midlands, UK; 3 Department of Oncology, UCL, London, UK

## 

Most childhood cancers are rare conditions, so research needs to be efficient. With only a limited number of children available for trials per year it seems sensible to use data from previous research. Systematic review methodology can do this whilst minimizing bias. Others also recommend undertaking a systematic review as part of a trial planning process in order to "reduce unwanted duplication, help ensure that new research builds on lessons from earlier research and place the findings of the new research in proper context". [Clark M 2007] However, there are some who would question the value of undertaking systematic reviews when only less than optimally designed studies are available, which is particularly true in paediatric oncology where non-comparative studies dominate.

Using a recently completed systematic review that investigated the effectiveness of ^131^I-meta iodobenzylguanidine (^131^I-mIBG) molecular radiotherapy for neuroblastoma, we aim to present some of the methodological challenges that we encountered during the review, such as study identification, quality assessment and "salami" publications. We will also discuss the presentation, analysis and interpretation of the results. We chose to perform quantitative analyses, which gave estimates of effect sizes, with measures of uncertainty, and evidence on dose/response relationships. We considered that such analyses gave greater insight into the data as a whole, while also being aware that quantitative analyses of generally poor quality data might be seen as providing a false sense of validity to sub-optimally designed studies.

